# Sequencing the Connectome

**DOI:** 10.1371/journal.pbio.1001411

**Published:** 2012-10-23

**Authors:** Anthony M. Zador, Joshua Dubnau, Hassana K. Oyibo, Huiqing Zhan, Gang Cao, Ian D. Peikon

**Affiliations:** 1 Cold Spring Harbor Laboratory, Cold Spring Harbor, New York, United States of America; 2 Watson School for Biological Sciences, Cold Spring Harbor, New York, United States of America

## Abstract

Connectivity determines the function of neural circuits. Historically, circuit mapping has usually been viewed as a problem of microscopy, but no current method can achieve high-throughput mapping of entire circuits with single neuron precision. Here we describe a novel approach to determining connectivity. We propose BOINC (“barcoding of individual neuronal connections”), a method for converting the problem of connectivity into a form that can be read out by high-throughput DNA sequencing. The appeal of using sequencing is that its scale—sequencing billions of nucleotides per day is now routine—is a natural match to the complexity of neural circuits. An inexpensive high-throughput technique for establishing circuit connectivity at single neuron resolution could transform neuroscience research.

## Abstract 2

Recasting the study of neural circuitry as a problem of high-throughput DNA sequencing instead of microscopy holds the potential to increase efficiency by orders of magnitude.

Neuroscientists seek neural explanations of perception, thought, and behavior. What does such an explanation look like? One of the earliest examples is Descartes' account [1] of the reflex withdrawal of a foot from a fire (Figure 1A). Descartes hypothesized that small particles of the fire displace the skin of the foot, which pulls on a tiny thread and thereby opens a pore in the pineal gland, releasing animal spirits, which flow back via a hollow tube into the foot to cause retraction. Although more modern accounts of the spinal reflex arc differ in important mechanistic and anatomical details, the kernel of Descartes' explanation is both correct and intellectually satisfying: the neural circuit he describes immediately implies the causal relationship between the stimulus and the resultant action. Circuit-level explanations of computation and behavior represent the gold standard.

**Figure 1:**
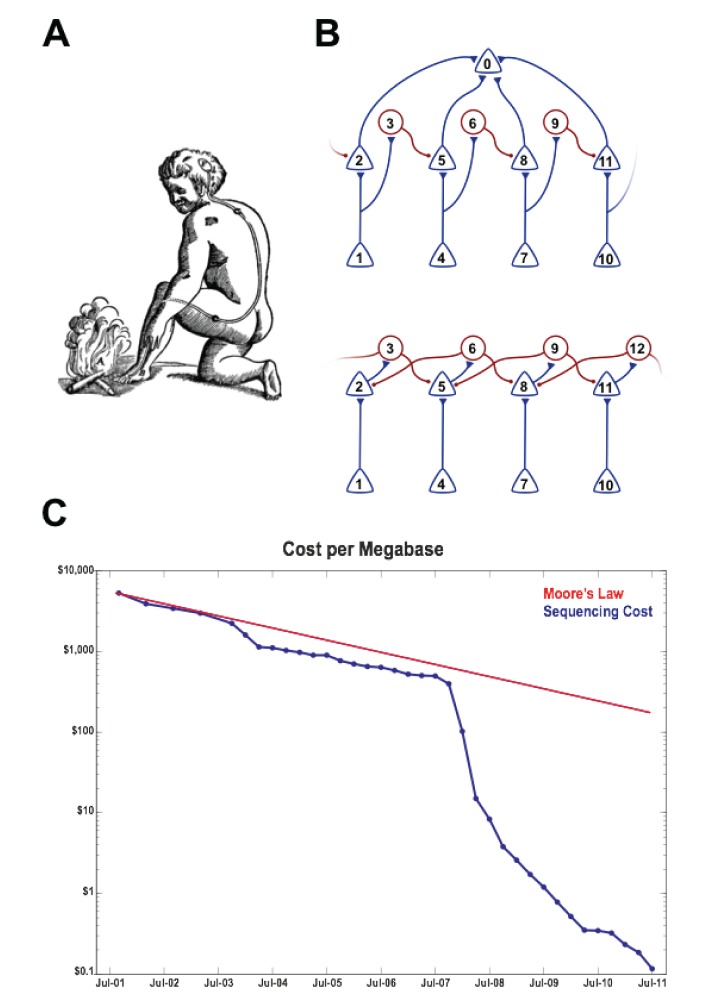
The wiring of neural circuits is highly structured. (A) Descartes' model of the foot withdrawal reflex. (B) Two similar circuits in which the computation is readily deduced from the wiring. The circuit on the top is directionally selective, whereas the one on the bottom performs a center-surround computation. (C) The costs of DNA sequencing are falling exponentially. From 2001 to 2007, the costs of sequencing dropped exponentially, in pace with Moore's law [18] for computation. Since the introduction of “next generation” sequencing technologies in 2008, the cost of sequencing has fallen more than 10-fold every year, compared with the steady 1.4-fold yearly drop for computing power. Data from http://www.genome.gov/sequencingcosts/.

## Why Is the Single Neuron Connectome Important?

Connectivity can be studied at different spatial scales. Conventional neuroanatomical methods probe the connectivity between brain regions. Such analysis reveals, for example, that the retina is connected to the visual thalamus, which in turn is connected to the visual cortex. The importance of mesoscopic connectivity in the mammalian brain is uncontroversial—different brain areas represent different kinds of information and have clearly distinct functions, so it is easy to see how knowing the connections among areas at the mesoscopic level will be useful. There are currently several major efforts to describe systematically the mesoscopic-scale connectivity of the mouse, macaque, and human brain [2].

Mesoscopic connectivity represents the natural anatomical complement to conventional physiological approaches, such as extracellular recording, for studying how populations of neurons encode information and control behavior. However, such physiological approaches tend to obscure the identity of the neurons under study. From the point of view of conventional extracellular recording, neurons within a brain area (e.g., visual area MT) differ only by their responses to sensory inputs and other external variables. Indeed, in physiological studies neurons are often referred to as interchangeable “units”; differences among nearby neurons are often attributed to random variation. Such assumptions are often incorporated into theoretical models, in which it is often assumed that cortical wiring is random, and therefore, only the statistical properties of neural connections, such as the average number of inputs per neuron, need be specified [3],[4]. In the absence of data about the relationship between the function of a neuron and its position within the local circuit, a description of connectivity at the mesoscopic level may seem sufficient.

The circuits in Figure 1B illustrate how connectivity beyond the mesoscopic—at the level of synaptic contacts between pairs of individual neurons—can also be useful. In the motion detection circuit on the top, sequential activation of input neurons from left to right (1, 4, 7, 10) will generate less activity in the output neuron (0) than activation from right to left (10, 7, 4, 1). The lateral inhibition circuit on the bottom is wired similarly, but the addition of a few extra inhibitory connections renders it insensitive to directional motion. These simple examples reveal how detailed connection information can provide immediate insight into the computations a circuit performs and can generate hypotheses that can be tested physiologically.

In practice, most computations are not understood at this level of precision. In part the reason is simply that detailed circuit information is largely unavailable. Indeed, the complete wiring diagram, or “connectome,” is known for only a single nervous system, that of the tiny worm *C. elegans*, with 302 neurons connected by about 7,000 synapses [5],[6]. Interestingly, the utility of the connectome in *C. elegans* is somewhat limited because function is highly multiplexed, with different neurons performing different roles depending on the state of neuromodulation [7], possibly as a mechanism for compensating for the small number of neurons.

Mammalian circuits contain orders of magnitude more neurons than *C. elegans*. Although neuromodulation is important in mammalian circuits, the need to multiplex function may not be as severe as in *C. elegans*, which may render the relationship between circuitry and function more transparent. In mammals there is ample evidence that the connectivity of a neuron correlates with its function. For example, whether a neuron in primary visual cortex is simple or complex is correlated with cell layer; cell layer is in turn a surrogate for connectivity. Even more striking is the finding that neurons in primary visual cortex that project to the motion-sensitive area MT represent a homogenous population whose motion sensitivity is more similar to that of neurons in MT than to other V1 neurons [8]. Observations such as these reinforce the notion that connectivity predicts function.

## Current Approaches to the Connectome

There are currently two main approaches to determining single cell connectivity. The first is based on physiology. This approach can be quite powerful and has yielded tantalizing evidence of the precise nature of connectivity of the cortical circuits. In one series of experiments, Callaway and colleagues used laser scanning photostimulation to probe connectivity in visual cortex [9]. They found that if two nearby neurons in layer 2/3 are connected, then they share input from single neurons in layer 4, but if they are not connected they do not share input. Thus, the input from layer 4 to layer 2/3 appears to consist of at least two independent “subnetworks,” which happen to overlap in space. In a different set of experiments, Chklovskii and colleagues [10] used whole cell methods to assess connectivity among triplets of neurons. By enumerating all 16 possible ways that three neurons can be connected, they discovered that several connectivity motifs were overrepresented above the chance levels predicted by the pairwise connection probabilities. Thus connectivity among triplets of cortical neurons deviates markedly from the null hypothesis of random connectivity. Unfortunately, physiological approaches do not readily scale up to an entire brain. Nevertheless, findings such as these hint at the rich structure yet to be uncovered in cortical circuits and motivate the development of higher throughput technologies.

The second approach is based on electron microscopy (EM). EM is required because light microscopy does not have sufficient resolution to establish whether two nearby neuronal processes are merely close or whether they have actually formed a synapse. Reconstruction of serial electron micrographs has yielded what to date is the only complete connectome, that of *C. elegans* [5],[6]. However, even for this simple nervous system, the reconstruction required a heroic effort—over 50 person-years of labor to collect and analyze the images. The difficulty of EM-based reconstruction arises from the fact that stacks of many individual images need to be aligned to track each axonal or dendritic process back to the soma; misalignment of even a single pair of images can result in an error in the wiring diagram, rendering the reconstruction of long-range connections particularly challenging. It is a testament to the importance of the connectivity problem that several research groups have made remarkable progress in automated EM reconstruction [11]–[13].

Several recent technical advances raise the possibility that a third class of approaches, based on light microscopy, may succeed in mapping circuit connectivity. *GRASP* (“GFP Reconstituted Across Synaptic Partners”) [14],[15] allows synaptic contacts to be resolved at the level of light microscopy. *Brainbow* [16] can be used to trace axons and dendrites over considerable distance. This technique relies on stochastic and combinatorial expression of several fluorophores (XFPs). Each neuron expresses a random collection of up to four different XFPs in different ratios, to achieve a theoretical palette of more than 100 different colors. The randomization is achieved by clever application of Cre-lox recombination, wherein the protein Cre recombinase catalyzes the inversion or excision of DNA between a pair of short (34 nucleotide) sequences termed lox sites. Finally, it is now possible to image an entire mouse brain using two-photon microscopy in hours or days [17]. Although these advances highlight the considerable promise of light microscopy for mapping neural circuits, such approaches are likely to be limited to sparse networks.

## DNA Sequencing as a Novel Method of Solving the Connectome

Here we propose to exploit high-throughput DNA sequencing to probe the connectivity of neural circuits at single-neuron resolution. Sequencing technology has not previously been applied in the context of neural connectivity, but the sequencing approach has tremendous potential. The advantage of sequencing is that it is already fast—sequencing billions of nucleotides per day is now routine—and, like microprocessor technology [18], getting faster exponentially. Moreover, the cost of sequencing is plummeting (Figure 1C): it currently costs less than $5,000 to sequence an entire human genome, and the race is on to reach the $1,000 genome. Thus, by converting brain connectivity from a problem of microscopy to a problem of sequencing, it becomes tractable using current technology.

**Figure 2:**
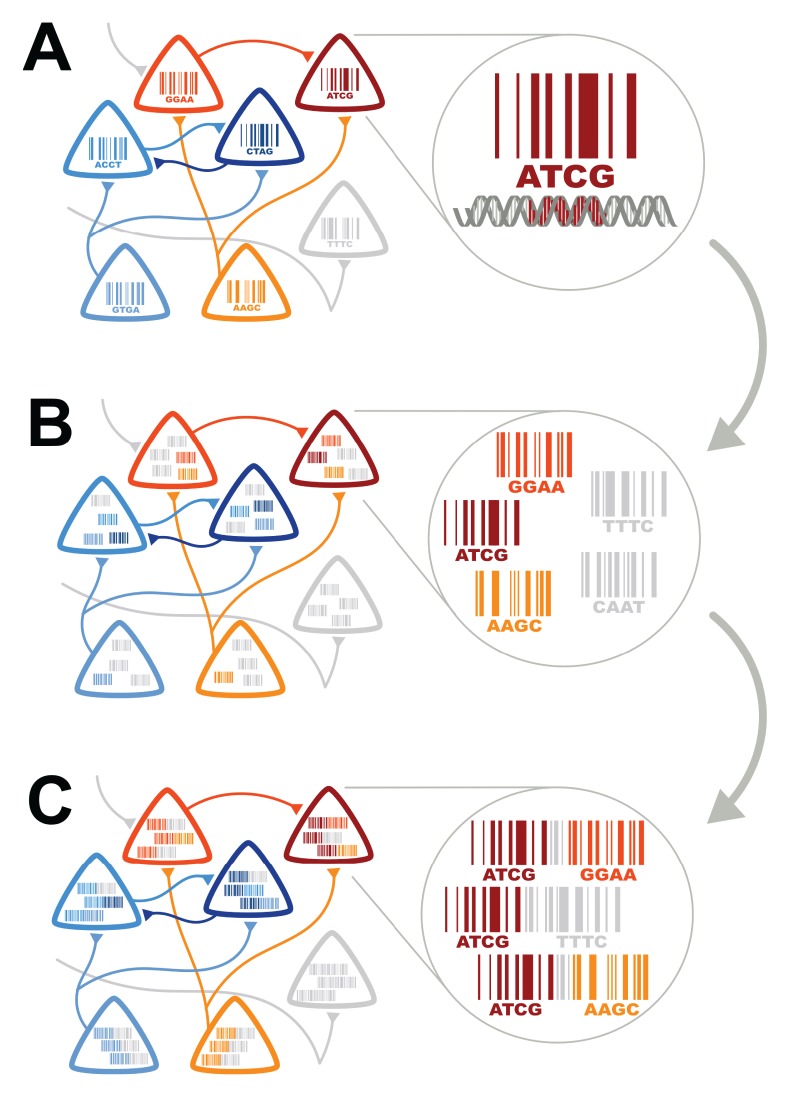
Converting connectivity into a sequencing problem can be broken down conceptually into three components. Each component of BOINC has many possible implementations. (A) First, each neuron must be labeled with a unique sequence of nucleotides—a DNA “barcode”. (B) Second, barcodes from synaptically connected neurons must be associated with one another, so that each neuron can be thought of as a “bag of barcodes”: copies of its own “host” barcode and copies of “invader” barcodes from synaptic partners. (C) Finally, host and invader barcodes must be joined into barcode pairs. These pairs can be subjected to high-throughput sequencing.

**Figure 3:**
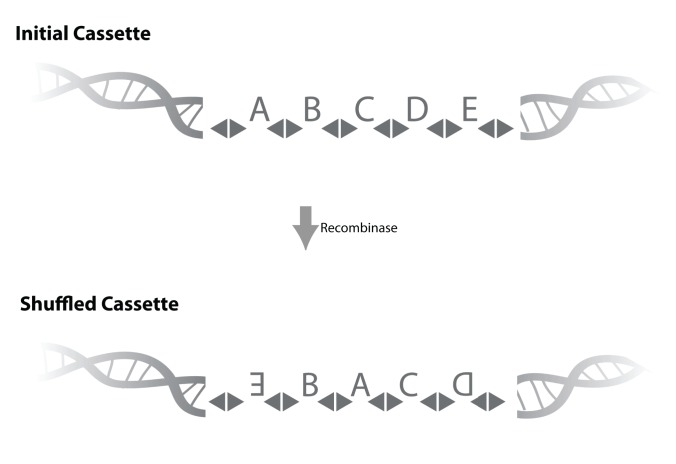
In vivo barcode generation. One strategy for generating sufficient diversity to barcode every neuron's DNA uniquely is shown above. In this strategy, inspired by Brainbow [16], each cell's genome contains a cassette consisting of a sequence of short unique barcode elements A…E… (top). Each barcode element is flanked by recombination sites (triangles). Upon expression of a suitable recombinase, these barcode elements shuffle and invert (shown here by inverted letter). The theoretical diversity that can be generated by this is *2^N^N!*, where *N* is the number of barcode elements. For a cassette containing *N* = 12 elements, the theoretical diversity is 2×10^12^, far more than needed to barcode the 10^8^ neurons in a mouse brain uniquely. Note that if a conventional recombinase like cre or flp is used here, excision will dominate over inversion and the resulting diversity scales with the number of barcode elements *N*. To avoid excision we use RCI [24], a recombinase that inverts but does excise.

BOINC, the method we propose for converting connectivity into a sequencing problem, can be broken down conceptually into three components (Figure 2). First, each neuron must be labeled with a unique sequence of nucleotides—a DNA “barcode” (Figure 2A; see also Figure 3). The requisite barcoding is conceptually similar—though different in detail—to the generation of antibody diversity by B cells in the immune system through somatic recombination. The idea of barcoding individual neurons is inspired by Brainbow, except that here DNA sequences substitute for fluorophores (XFPs). The advantage of using sequences is diversity: whereas Brainbow allows for at most hundreds of color combinations, a barcode consisting of even 20 random nucleotides can uniquely label 4^20^ = 10^12^ neurons, far more than the number of neurons (<10^8^) in a mouse brain.

Second, barcodes from synaptically connected neurons must be associated. One way to associate a pre- and postsynaptic barcode is by means of a transsynaptic virus such as rabies [19] or pseudorabies (PRV) [20]. These viruses have evolved exquisite mechanisms for moving genetic material across synapses and have been used extensively for tracing neural circuits in rodents. To share barcodes across synapses, the virus must be engineered to carry the barcode within its own genetic sequence. After transsynaptic spread of the virus each postsynaptic neuron can be thought of as a “bag of barcodes,” consisting of copies of its own “host” barcodes, along with “invader” barcodes from presynaptically coupled neurons (Figure 2B).

**Figure 4:**
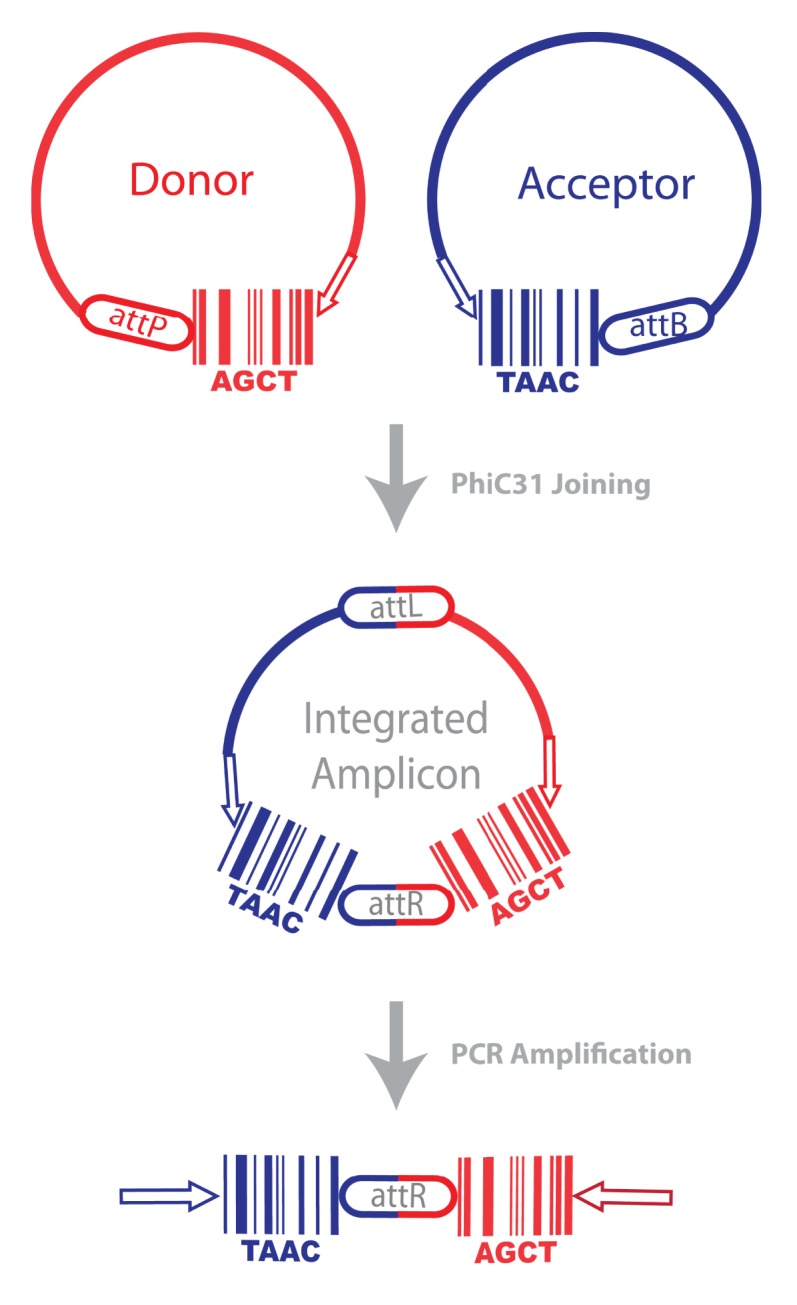
Joining barcodes with phiC31 integrase. One strategy for joining barcodes is based on phiC31 integrase [25]. PhiC31 mediates the integration of a 35-nucleotide AttB site with a 35-nucleotide AttP site to form an AttL and an AttR site. Because the AttL and AttR sites are not targets of phiC31, this reaction is irreversible (unlike comparable reactions with cre and flp). Once the barcodes are joined, they can be amplified by PCR (using primers complementary to the arrows) for sequencing.

Finally, barcodes from synaptically connected neurons must be joined into single pieces of DNA for high-throughput sequencing (Figure 2C; see also Figure 4). Barcodes are joined in vivo, so there is no need to isolate individual neurons prior to extracting DNA. Since only those pairs associated in vivo are actually joined, observing a host-invader barcode pair indicates that the host and the invader were synaptically coupled. For example, if upon sequencing we observe host barcode D with invader barcodes B and C, we can infer that neuron D is connected to neurons B and C.

**Figure 5:**
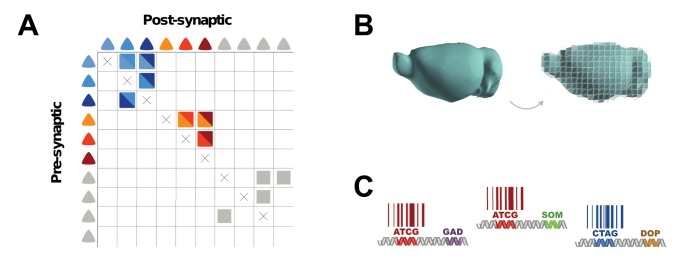
Beyond the abstract connectivity matrix. (A) The connectivity of the circuit obtained through sequencing can be read out by filling in the entries of a (sparse) connectivity matrix based on which host-invader barcode pairs that were found by sequencing to be joined together. (B) The sequencing approach can be extended to recover information about brain region. To associate each barcode with a specific brain region, the brain can be sectioned prior to extracting barcodes. The DNA extracted from each section can be sequenced separately, or DNA from multiple regions can be pooled after adding a DNA tag to each region. The size of the sections determines the spatial resolution to location of each barcode; a resolution of a few hundred microns could be easily achieved and would suffice for many purposes (e.g., to distinguish nearby structures such as auditory and visual cortex). (C) The sequencing approach can be extended to recover information about brain region and cell type. To make inferences about the cell type from each barcode that arose, mRNA transcripts from each cell can be barcoded (e.g., by RNA transsplicing [26]). Thus, if barcode 242 were found tagging both GAD-67 and parvalbumin, neuron 242 would likely be a fast-spiking GABAergic interneuron.

Since most neurons are only sparsely connected to other neurons in the brain—for example, in the mouse cortex a typical neuron is connected with perhaps 10^3^ of its 10^8^ potential partners—only a small subset of the potential host-invade barcode pairs will actually be observed. Thus upon high-throughput sequencing, we can fill in the non-zero elements of the sparse connectivity matrix (Figure 5A).

In its simplest form the sequencing approach yields only a connectivity matrix. Missing from this matrix are at least two kinds of useful information typically obtained with conventional methods based on microscopy: information about the brain region (e.g., primary auditory cortex, striatum, etc.) from which each barcode originates (Figure 5B), and information about the cell type (e.g., dopaminergic, fast-spiking GABAergic, etc.) of each barcoded neuron (Figure 5C). However, several strategies can be used to augment the connectivity matrix with both kinds of information. Thus, as sequencing-based connectivity analysis matures, it may generate a view of connectivity similar to that provided by traditional methods.

In summary, there are three technical challenges that must be overcome to map neural circuits using high-throughput sequencing: (1) barcoding each neuron, (2) associating barcodes from connected neurons, and (3) joining the barcodes prior to sequencing. We are developing an approach based on PRV amplicons [21]. Although there are many technical problems, including PRV toxicity and monosynaptic spread [19], which need to be addressed, this approach promises to offer a proof of principle for our proposal to convert connectivity into a sequencing problem.

## Costs

In the 2 and half years between the introduction of “next generation” DNA sequencing technologies in January 2008 to the most recent data in July 2011, the cost of sequencing fell by a factor of 1,000 (Figure 1C). This 15-fold yearly rate of improvement far exceeds even Moore's law, according to which computer costs drop 2-fold every 2 years. Just as Moore's law drove and was driven by the computer revolution, so the drop in sequencing costs is driven by the prospect of a genomics revolution in medicine. Although such a precipitous rate of improvement of sequencing cannot be sustained indefinitely, it would not be surprising if commercial pressures were to drive costs down by another factor of 100 or moreover the next few years.

How much would it cost to “sequence the cortex” of a mouse? We can put a lower bound on the current sequencing cost as follows. The mouse cortex consists of about 4×10^6^ neurons [22]. Suppose that each cortical neuron connects to about 10^3^ other cortical neurons, so that there are 4×10^6^×10^3^ = 4×10^9^ connections. If we assume that each barcode is 20 nucleotides, then we have 4×10^9^ connections×20 nucleotides/barcode×2 barcodes/connection = 1.6×10^11^ nucleotides. Assuming that the fraction of unsampled connections is exp(−k/N), where *k* is the number of reads and *N* is the number of barcodes, then with 3-fold oversampling (4.8×10^11^ nucleotides) we would expect to sample 95% of connections. At $0.1/10^6^ nucleotides (July 2011), this would cost $48,000 and could easily drop several orders of magnitude in a few years. A similar calculation for *Drosophila*, with 10^5^ neurons and 10^7^ connections, yields $1/brain; and for *C. elegans*, with 302 neurons and 7,000 connections, sequencing costs would be essentially negligible. Although these are best case estimates and do not include costs other than sequencing, the possibility of a $1 *Drosophila* connectome, or a $1,000 mouse cortical connectome, emphasizes the promise of recasting neural connectivity as a sequencing problem.

## Advantages and Limitations of the Sequencing Approach

Like any method, the sequencing approach is subject to false positives (i.e., inferred connections that do not exist) and false negatives (actual connections that are missed). Although the prevalence of each type of error will depend on the details of the implementation, with the sequencing approach most errors will likely be false negatives. Possible sources of false negatives include failure of transsynaptic barcode transport and undersampling of the amplified barcode pairs. Most sequencing errors will also result in false negatives, but these can be minimized by judicious design of the barcodes. Possible sources of false positives include loss of synapse specificity in the transsynaptic transport of barcodes and insufficient diversity in the pool of possible barcodes. By contrast, false positives are likely to be an important source of error in microscopy-based approaches in which inaccurate tracing of a neuronal process across tissue sections can lead to misattribution of a synaptic connection to the wrong parent.

The sequencing approach provides different information from conventional microscopy-based approaches. Electron microscopy provides a wealth of data not available in the sequencing approach, including information about neuronal morphology, as well as about the subcellular placement, number, and size of synapses. On the other hand, the sequencing approach has the potential to provide direct access to the molecular expression profile of individual neurons—whether it is dopaminergic or expresses a marker such as parvalbumin that tags the neuron as belonging to a particular subtype of interneuron. Moreover, with the sequencing approach, local and long-range connections are equally accessible; by contrast, with microscopy the probability of inaccurately tracing a synaptic connection increases with distance, rendering the reconstruction of inter-areal connections a particular challenge.

## Conclusions and Perspectives

The appeal of the sequencing approach rests in its promise of high throughput, as defined by cost and mapping time. Low-cost sequencing of brain circuits could be used as a screening test to generate hypotheses about how circuits change with development, learning, genetic manipulations, or any other experimental factor. For example, autism has been hypothesized to arise from genetic lesions that disrupt local and long-range connectivity, but different autism candidate genes may disrupt circuits differently [23]. High-throughput circuit screening would enable a systematic comparison of the similarities and differences among brain circuits in animal models of autism. A high-throughput circuit screen has the potential to transform how experiments are designed.

What will we learn from sequencing the connectome? Perhaps it is instructive to turn to the lessons learned from sequencing the human genome. Knowledge of the complete genome provides the starting point for much of modern biological research, transforming how research is conducted in the post-genomic era. A cheap and rapid method for deciphering the wiring diagram of an entire brain may have a comparably profound impact on neuroscience research.
